# Proseek single-plex protein assay kit system to detect sAxl and Gas6 in serological material of brain tumor patients

**DOI:** 10.1016/j.btre.2018.e00252

**Published:** 2018-04-17

**Authors:** Heidi Jaksch-Bogensperger, Anna Hammerschmid, Ludwig Aigner, Eugen Trinka, Renate Gehwolf, Yvonne Ebner, Markus Hutterer, Sebastien Couillard-Despres

**Affiliations:** a University Hospital for Obstetrics and Gynaecology, Paracelsus Medical University Salzburg, Müllner Hauptstrasse 48, A-5020, Salzburg, Austria; b Institute of Molecular Regenerative Medicine, Spinal Cord Injury and Tissue Regeneration Center Salzburg, Paracelsus Medical University, Strubergasse 21, A-5020, Salzburg, Austria; c Institute of Experimental Neuroregeneration, Spinal Cord Injury and Tissue Regeneration Center Salzburg, Paracelsus Medical University, Strubergasse 21, A-5020, Salzburg, Austria; d University Hospital of Neurology, Christian-Doppler-Klinik, Paracelsus Medical University Salzburg, Ignaz-Harrer-Straße 79, A-5020, Salzburg, Austria; e Institute of Tendon and Bone Regeneration, Spinal Cord Injury and Tissue Regeneration Center Salzburg, Paracelsus Medical University, Strubergasse 21, A-5020, Salzburg, Austria; f University Hospital of Psychiatry, Psychotherapy and Psychosomatic, Paracelsus Medical University Salzburg, Ignaz-Harrer-Straße 79, A-5020, Salzburg, Austria; g Department of Neurology 1 – Neuromed Campus, Kepler University Hospital Linz, Wagner-Jauregg Weg 15, A-4020, Linz, Austria

**Keywords:** High-grade glioma (HGG), sAxl, Gas6, Proximity extension assay (PEA), Proseek, Serum

## Abstract

• The receptor tyrosine kinase (RTK) Axl and its ligand Gas6 are critically involved in the pathogenesis of high-grade glioma (HGG). Both proteins were found to be overexpressed e.g. in tumor cells, mediating cell proliferation and migration as well as tumor angiogenesis and neuroinflammation. The extracellular domain of Axl (sAxl) and Gas6 were found in the peri-tumoral edema and blood of animals as well as in human glioma tissue. Therefore, we monitored the level of sAxl and Gas6 in human blood samples. To increase the sensitivity of protein detection beyond commonly used standard methods we preliminary tested the innovative Proseek Single-Plex Protein Assay Kit System from Olink Bioscience together with new antibodies against the soluble RTK sAxl and its ligand Gas6. We conclude that the Proseek method is a highly sensitive and fast procedure that can be used as a possible powerful tool compared to routinely used ELISA-methods.

## Highlights

• Establishment of an alternative method beside routinely used ELISA to measure levels of sAxl and Gas6 in serological material of HGG patients is proposed.
• Both antibodies are established with the powerful combination of protein detection and PCR amplification using the Proseek Single-Plex Assay.
• This tool quantifies single proteins in solution with a maximum of sensitivity and specificity to visualize low levels of proteins in just 1 μl blood sample within one day.

## 1 Introduction

High-grade glioma (HGG), WHO grade III-IV, are the most common primary brain tumors in adults with an incidence of 6–8/100.000 inhabitants per year. Within this group, glioblastome multiforme (GBM) WHO IV is the most aggressive subtype and represents the biggest group therein. Anaplastic glioma WHO grade III imply astrocytoma and oligodendroglioma [1,2]. The current standard treatment of GBM includes a maximal surgical tumor resection, followed by radio- and chemotherapy with temozolomide [3,4].

Since the receptor tyrosine kinase (RTK) Axl and its ligand growth arrest specific gene 6 (Gas6) are known to be co-expressed in HGG tissue correlating with a poor prognosis [5], we were interested on testing a new adapted open format reagent kit method system from Olink Bioscience called Proseek Single-Plex Protein Assay measuring the soluble (extracellular) portion of the Axl RTK (sAxl). The usual method of choice to measure levels of sAxl and Gas6 in human sera is a sandwich enzyme-linked immunosorbent assay (ELISA), which general protocol has been optimized recently based on challenging stability and storage conditions, but also masking effects of unknown components in serum [6]. Since ELISA measurement includes time-consuming washing procedures, the Proseek Single-Plex Assay provides results within 24 h without washing steps. Furthermore, only 1 μl of serum, plasma, or almost any other type of biological sample such as liquor, is sufficient for valide results. Another interesting part of the method is the combination of oligonucleotide-labeled antibodies (binding the target protein within the sample) and the formation of a new PCR target sequence, which can be quantified by standard real-time PCR. This powerful combination of protein detection and PCR amplification to quantify single proteins in solutions with a maximum of sensitivity and specificity is able to visualize low levels of proteins even before tumors can be detected by imaging technologies prior therapy and also should help for therapy monitoring. Therefore we were interested on the combination of this method with targeted human protein biomarkers for tumor vascularization, sAxl and Gas6.

The receptor tyrosine kinase Axl, beside Tyro3/Sky and Mer (syn. TAM family), is characterized by an extracellular domain (ECD) of two immunoglobuline-like domains abreast of two fibronection-type III domains [7,8].

After ligand binding (e.g. Gas6, Protein S), the tyrosine kinase domain of the receptors of the TAM family activates the intracellular signal transduction downstream by receptor dimerisation and initiation of autophosphorylation [9,10]. Axl/Gas6 signaling can induce apoptosis inhibition in a broad range of cells, [[11], [12], [13], [14]] and is involved in cell migration, essential for tumor invasiveness, metastasis and neoangiongenesis. Therefore the Axl/Gas6-system is involved in various physiological processes, including angiogenesis and several types of human cancer [5,9,10,15,16].

## 2 Material and methods

### 2.1 Study population and sample collection

To generate the new method to measure the detection of sAxl and Gas6, 3 patients of ≥18 years and ≤80 years of age with the diagnosis of a first or second recurrent or progressed high-grade glioma measured by standard MRI were included. For comparison, the serum of a healthy 25-year old woman was added (control). Sera of patients with recurrent or progressive HGG receiving anti-angiogenic bevacizumab therapy (P02: female, age 47; P04: male, age 55; P05: male, age 42) were used. After baseline and treatment start with Avastin, clinical follow ups started with serum sample collections at week 1 ± 1 day, followed by clinical follow up visits at week 2. Afterwards every second week until clinical progression (e.g. P01 S1: patient 01 with serum sample S1 of the first week, S2 of the second week, and samples S3 to S7 for every second week), serum samples were collected and used for measurements. Ethical approval for this study was granted by the local research ethics committee (AM3752_LEK).

### 2.2 Sample preparation

For serum sample preparation, standard laboratory tests including chemistry (differential (%), coagulation, biochemistry) and haematology panels have been performed. At baseline evaluation a serum pregnancy test (female patient with reproductive potential) and a test for infections (Hepatitis B/C, HIV) have been carried out. All patients have been consented for the collection and storage of blood (University Hospital of Neurology, PMU, Salzburg), and markers have been evaluated using above-mentioned method. At baseline and during periodic follow-up visits in the course of an antiangiogenic treatment with Avastin (Bevacimzumab 10 mg/m2 body surface every two weeks), serum samples have been collected at baseline, in the first and second week and afterwards every 2 weeks (S1, S2, S3,…) until tumor progression for precise Proseek analysis. Therefore one serum tube derived from vein puncture for testing the combination of the new markers associated with tumor neovascularization (sAxl, Gas6) by Proseek-method have been used. Therefore blood samples were let for 10 min at room temperature and centrifuged for 10 min at 1408 × *g*. Aliquots of the supernatants were frozen to −80 °C. Serial dilutions for the proteins sAxl and Gas6 were prepared as a positive control and to establish a calibration curve. To this end, the recombinant and affinity purified Axl (DY154, R&D systems) and Gas6 (DY885, R&D systems) have been diluted in Calibrator Diluent as follows: Axl 139 ng/ml, 100 ng/ml, 10 ng/ml, 1 ng/ml, 100 pg/ml, 10 pg/ml, 1 pg/ml, 0.1 pg/ml, 0.01 pg/ml, 0 (Calibrator Diluent); Gas6 230 ng/ml, 100 ng/ml, 10 ng/ml, 1 ng/ml, 100 pg/ml, 10 pg/ml, 1 pg/ml, 0.1 pg/ml, 0.01 pg/ml, 1 fg/ml, 0 (Calibrator Diluent).

### 2.3 Proseek-proximity extension assay (PEA) technology

Proseek is a reagent Kit system from Olink Bioscience (Uppsala, Sweden) to detect and quantify proteins in sample material like serum and plasma, based on the Proximity Extension Assay (PEA) technology. The PEA is a method for single protein detection based on a Proximity-dependent DNA polymerization event. It can be performed using 2 Proximity probes. For this project, we took two polyclonal antibodies already established in ELISAs, but not for the Proseek reagent Kit system, to detect sAxl (AF154, R&D systems) and Gas6 (AF885, R&D systems). The Proseek Assay Development kit has been performed as described in the user manual version 3.0. First, the antibodies were conjugated either to the oligonucleotides (Oligonucleotide-labeled antibodies) A or B by using Proseek Probemaker A (Art.no. 93001-0010) or B (Art. No. 93002-0010) to create Proseek probes A and B. A dilution series of recombinant human sAxl (DY154, R&D systems) and recombinant human Gas6 (AF885, R&D systems) as antigen standard in Calibrator diluent (Proseek Assay Reagents, Art.no. 93003-1000) for standard curve was used. For negative control, Calibrator diluent without antigen was prepared for method application. The incubation of the dilution series and serum samples with Proseek probes A and B leads to the binding to the target protein. To dilute the Proseek probes and lower their real concentration, the Pre-Extension solution has been adducted before the Extension master mix has been added. Thereafter, the addition of a DNA polymerase will enable the extension of the hybridized oligonucleotides and the resulted sequence is detected and quantified by qPCR (real-time PCR amplicon, Proseek Assay Development Kit, Olink Bioscience).

### 2.4 Real-time based proseek technology

For this study, a two-step real-time PCR (qPCR) was performed by using the fluorophore FAM based on the TaqMan^®^ Protein Assays Probe Development Protocol (LifeTechnologies, Applied Biosystems, Austria). Proseek reagents have been prepared according to the Proseek user manual (Olink, Bioscience, 2014). Samples, buffer (recommended background control by the TaqMan^®^ Protein Expression Assay Protocol) or recombinant antibody for standard curve were incubated according to manual instruction. The PCR was performed using the TaqMan^®^ Protein Expression Assay reagent kit according to the protocol (also Master Mix without Polymerase). Data analysis and sample calculation were carried out using Microsoft Excel 2007. For the calibration curve of each test of sAxl and Gas6, the calibrators were measured in duplicates. Afterwards the cycle of quantification (Cq) was calculated as the mean value of 3 independent experiments and the background values of the control were subtracted. To assess the concentration of the proteins the formula y = kx + d has been used.

### 2.5 ELISA

Calibration curves using human sAxl (DY154, R&D Systems) and Gas6 (DY885, R&D Systems) were also analyzed using the DuoSet ELISA Development Kit for sandwich ELISA. Both Kit systems contain components required to measure natural and recombinant human Gas6 and sAxl according to the manufactures protocols. Detection was performed using a Siemens BEP III ELISA Reader and results have been processed using Microsoft Excel 2007. In detail, the capture antibody has been diluted in PBS and immediately coated on a 96-well microplate, á 100 μl and incubated at RT for ∼24 h. Each well has been washed 3-fold with 400 μl wash buffer by an autowasher and remained buffer has been removed by blotting the plate against paper towels. Plates were blocked in 300 μl adequate blocking buffer for 1 h at RT. In the meantime, samples and standards were diluted in the required concentrations. Plates were aspirated like before and samples and standards diluted in RD buffer were added (100 μl/well) and incubated for 2 h at RT. Afterwards, the aspiration step was repeated and 100 μl of the detection antibody per well was added for 2 h. Washing procedure followed, before the working dilution of streptavidin-HRP, 100 μl, for 20 min in the dark, starts. Plate has been washed as before and 100 μl substrate solution was added in the dark. 20 min later, the reaction has been stopped by adding 50 μl 2 N H_2_SO_4_ and determination of the optical density of each well has immediately been started at 450 nm.

## 3 Results

### 3.1 Establishment of the standard curve of Gas6 by using proseek-based PEA method

**Fig. 1:**
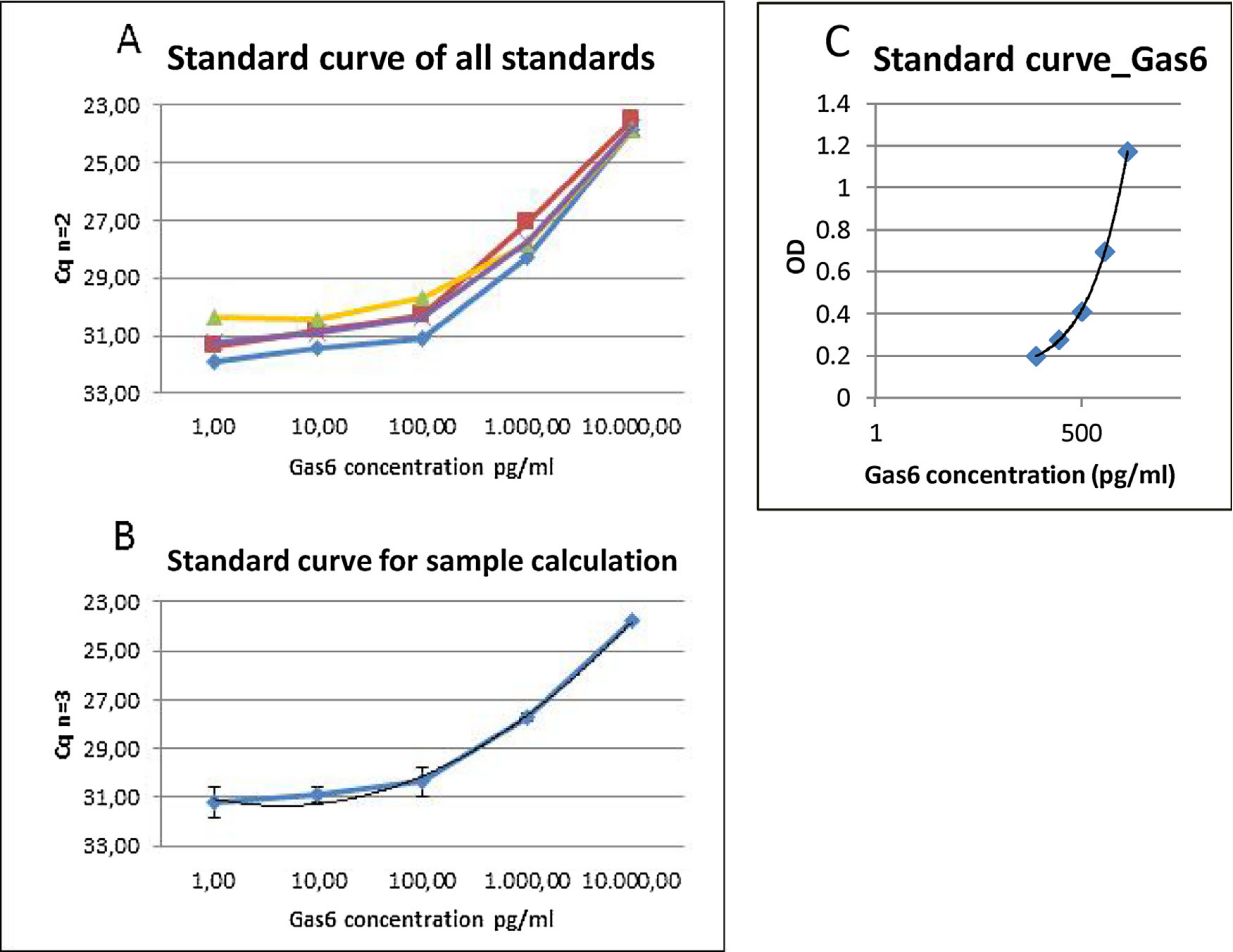
Calibration curve of Gas6 (AF885). Comparison of Proseek (A, B) and DuoSet ELISA Dy885 (C). Detection of each concentration of human recombinant Gas6 from 1 pg/ml to 10 000 pg/ml (n = 3). Bars represent standard deviations of the mean of 3 (A) independent measurements (duplicates) (B). Detection of Gas6 by ELISA Dy885 (C).

A linear serial dilution of a human Gas6 standard AF885 from R&D systems was generated between 1 pg/ml and 10 ng/ml and used for the amplification. The mean Cq value for each concentration standards was calculated based on measurements from three independent experiments performed in duplicates (n = 3; Fig. 1A, B). The calibrator diluent without antigen (Gas6) was subtracted from the measured standards of Gas6 to generate standard curve for further sample calculations. Using the same antibodies, performance of Gas6 detection using Proseek or ELISA were compared. Results revealed that the Proseek method was more sensitive to detect lower concentrations of Gas6 (1 pg/ml, 10 pg/ml, 100 pg/ml, 1 ng/ml, 10 ng/ml; Fig. 1B) as compared to the DuoSet sandwich ELISA method (2000 pg/ml, 1000 pg/ml, 500 pg/ml, 250 pg/ml, 125 pg/ml; Fig. 1C).

### 3.2 Proseek-based PEA method and establishment of the standard curve of sAxl

**Fig. 2:**
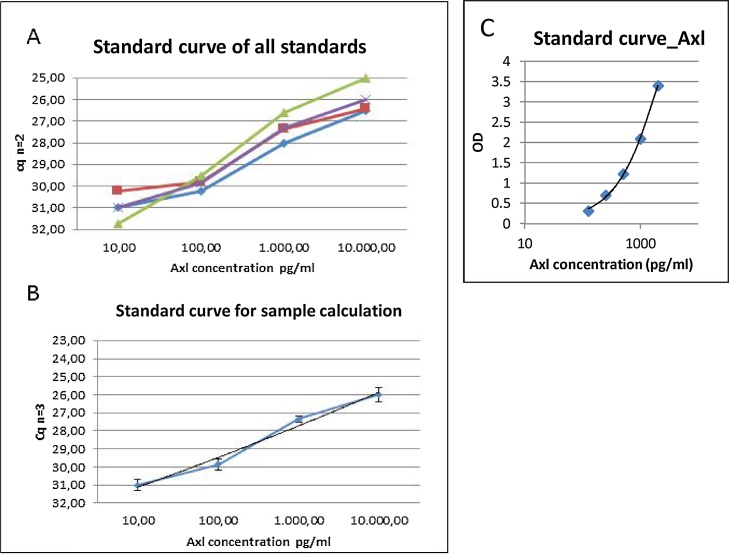
Calibration curve of Axl (AF154). Comparison of Proseek (A, B) and DuoSet ELISA DY154 (C). Detection of each concentration of human recombinant Axl from 10 pg/ml to 10 000 pg/ml (n = 3). Bars represent standard deviations of the mean of 3 (A) independent measurements (duplicates) (B). Detection of Axl by ELISA DY154 (C).

A linear serial dilution of a human sAxl standard (AF154, R&D systems) was generated between 10 pg/ml and 10 ng/ml and used for the amplification. The mean Cq value for each concentration standards was calculated based on measurements from three independent experiments performed in duplicates (n = 3; Fig. 2A, B). The polyclonal antibody directed against sAxl served as specificity control for the Proseek reaction in calibrator diluent. The calibrator diluent without antigen (sAxl) was substracted from the measured standards of sAxl to serve as standard curve for further sample calculations. Using the same antibodies, the performances of sAxl detections using Proseek or ELISA methods were compared.

Results revealed that the Proseek method was more sensitive to detect low concentrations of sAxl (10 pg/ml–10 ng/ml) as compared to ELISA detection (125 pg/ml–2 ng/ml) (Fig. 2C).

### 3.3 Detection of sAxl/Gas6 by using proseek-based PEA method in serological material

**Fig. 3:**
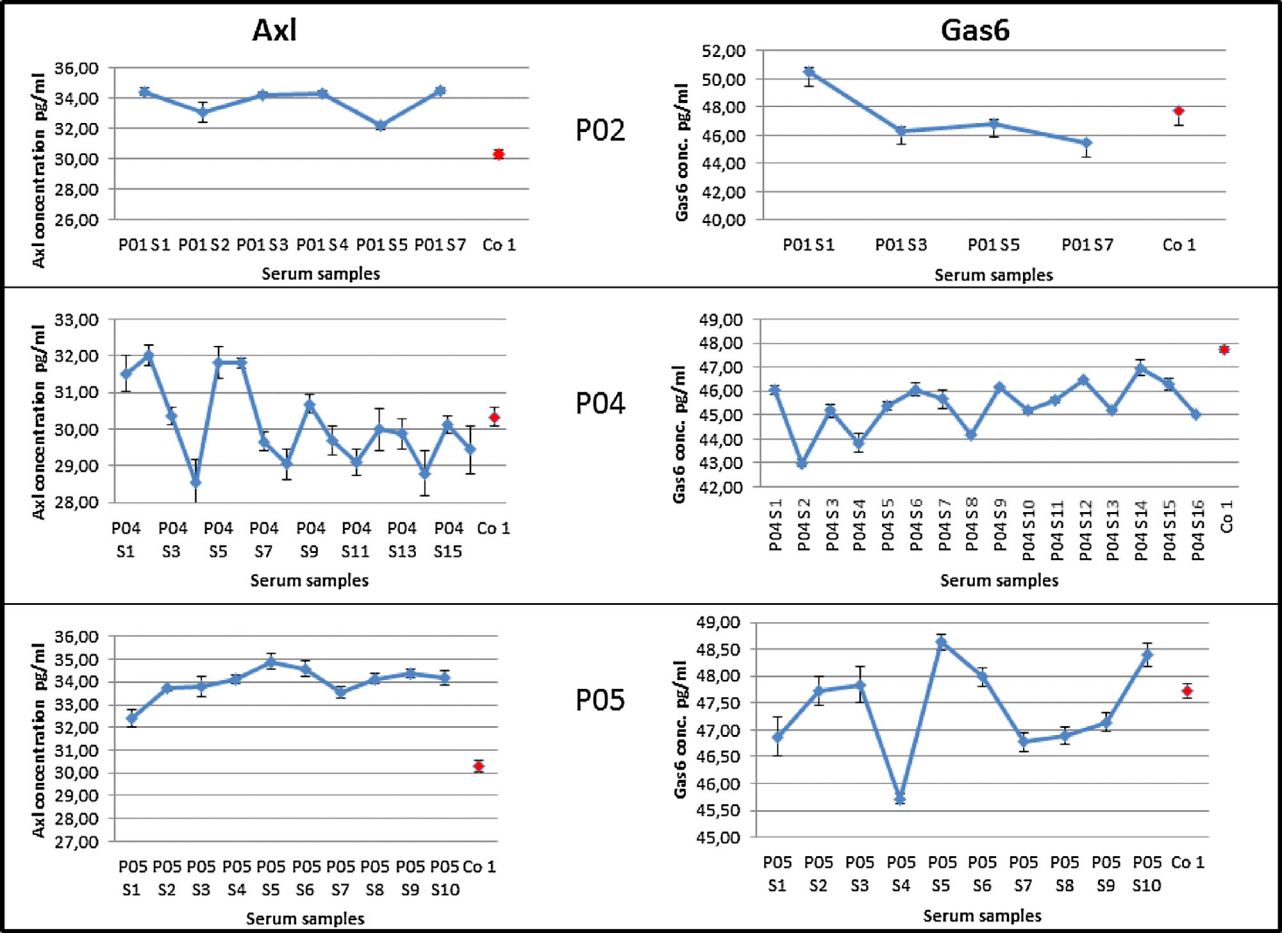
Concentrations of Axl and Gas6 in human serum samples during treatment by using Proseek. The calculations were performed according to the calibration curve in Fig. 1 for Gas6 and Fig. 2 for Axl using the formula y = kx + d. X-axis: P02, P04, P05 serum samples from human patients till recurrence (S). Red dot: serum of a healthy person. y-axis: protein concentration in pg/ml.

After establishing the standard curves for Gas6 (Fig. 1) and sAxl (Fig. 2), the concentrations of these proteins were determined within patients’ (P02, P04, P05) and a healthy control’s serums according the formula y = kx + d (Fig. 3). Measurements were performed in three independent experiments (n = 3) using the same polyclonal antibodies and the mean values were calculated. P02 and P05, but not P04, show elevated levels of the sAxl concentration in serum (>32 pg/ml) compared to a healthy person (<30.5 pg/ml). In P04, the sAxl concentration decreased after the second peak (S6) and reached a value comparable to the healthy person until S15 (Fig. 3). In general, all measurements reached a level of < = 100 pg/ml based on possible individual responses to therapy treatment and compatible for Proseek method (Fig. 3). Measurements of the Gas6 serum concentration was performed in 4 samples from P02, 16 from P04 and 10 from P05. No observable difference in Gas6 concentration between a healthy control (Co 1) and a HGG patient during therapy could be detected. Fig. 3 shows the mean values calculated from 3 independent experiments. The amplitudes of the signal were not balanced based on possible individual responses to therapy (Fig. 3).

## 4 Discussion

The aim of the study was the establishment of alternative methods beside routinely used sandwich enzyme-linked immunosorbent assay (ELISA) to measure levels of sAxl and Gas6 with the intention of developing a sensitive diagnostic instrument capable of detecting the growth and recurrence of HGG before the tumor reaches larger sizes required for direct visualization. Taken that sAxl and Gas6 plasma concentration are interesting parameters to identify different types of diseases such as HGGs, ELISA methods have been recently developed, allowing henceforth accurate analysis of sAxl in human serum for routine diagnostics [6]. The sAxl/Gas6 system is known to be expressed in HGG tissue and high levels correlate with a poor prognosis [5]. Therefore, we addressed the capacity of the PEA-based technique Proseek to improve the sensitivity of Axl/Gas6 detection in measurements performed before and during Bevacimzumab therapy above commonly used methods. Divers to the manufactures protocol for the Duoset ELISA Development Kit using sAxl (DY154, R&D Systems) and Gas6 (DY885, R&D Systems), the maximum standard concentration of 4000 pg/ml for saturation has been reached at 2000 pg/ml (Fig. 1, Fig. 2C). Additionally, the minimal standard concentration for both, sAxl and Gas6, using ELISA method was 125 pg/ml. In comparison, the PEA-based technique enables measurements of the standard concentrations for Gas6 from 1 pg to 10 ng/ml and for sAxl from 10 pg/ml to 10 ng/ml. These data suggest that PEA-based technology is very sensitive to detect Gas6 and sAxl in serum samples analyzed and that it allows to monitor concentrations of both proteins over time more efficiently than commonly used ELISA methods. Since we were interested to determine the changes and significances of higher plasma concentrations of sAxl and Gas6 in patients with HGG, we added a healthy person to provide reference values of physiological concentrations of both proteins, and to compare the individual course and response to treatment with Bevacimzumab.

The combination of DNA ligation and real-time PCR and the combination with PEA-established methods can be performed with standard laboratory equipment. The advantages of a PEA-based technique are that it is an elegant format and does not required time-consuming washing procedures leading to material loss. Moreover, measurements can be performed in sample volume as small as 1 μl, which is crucial for cases of precious biobanked material or paraffin-embedded tissues. Additionally, measurements were performed using commercially available polyclonal or monoclonal antibodies from R&D systems within 1 day. The advantage of using antibodies is that it is a powerful tool to combine protein detection with PCR amplification. The antibodies established for the Proseek Assay Development Kit are affinity-purified and have no BSA or other carrier proteins included that could interfere with conjugation (see AF885, DY254). After preparing the dilution series of the antigen standards (recombinant sAxl DY254, R&D systems; recombinant human Gas6, R&D systems), the antibody will bind to the target protein, the antigen. By extending the bonded probes with Oligos using Extension master mixes from the Kit through a DNA polymerization event, the real-time PCR amplicon has been created for PCR to increase sensitivity. The limitations of the assay are that the primary antibody has to be compatible and well selected which suits to the assay, e.g. R & D systems. Additionally the method needs some time for establishment to ensure precise detections (e.g. DNA ligation, real time-PCR). Also the needs of time has to be hold, especially for the time of binding to reduce unspecific clustering of proteins or due to low target antigen levels in body fluids (2 h room temperature or at +4 °C over night).

Further investigation is still needed to correlate the progression of sAxl and Gas6 concentrations in serum of HGG patients with disease progression, but Proseek Multi-Plex Protein Assay Kit system seemed to be a powerful tool to combine those 2 proteins for further analysis.
